# Vitiligo: A Narrative Review

**DOI:** 10.7759/cureus.29307

**Published:** 2022-09-18

**Authors:** Rutuja R Joge, Piyush U Kathane, Shiv H Joshi

**Affiliations:** 1 Community Medicine, Jawaharlal Nehru Medical College, Datta Meghe Institute of Medical Sciences (DU), Wardha, IND; 2 Community Medicine, Mahatma Gandhi Institute of Medical Sciences, Sevagram, IND

**Keywords:** autoimmune, skin color, hypopigmentation, vitiligo, pigmentation

## Abstract

Vitiligo, a common depigmenting cutaneous condition, is thought to affect 0.5%-2% of the world's population. During this condition, melanocytes are selectively lost, resulting in non-scaly, chalky-white macules. Achromic macules and patches are side effects of the multifaceted disease vitiligo, defined as the absence of epidermal pigmentation. The causes of this disaster are three significant factors. A suppressed reaction to touch allergens is one of many abnormal activities of the hypopigmented epidermis, which has also been observed in hypopigmented rats. The white epidermis of people with albinism, which is the same color as vitiligo, is more vulnerable to skin carcinoma; the white epidermis of people with vitiligo does not develop non-melanoma skin carcinoma. The overall etiology of vitiligo, which is now categorically recognized as an immunological illness, has made significant strides in recent years. Even though vitiligo is frequently dismissed as an esthetic issue, it can have serious mental consequences and significantly interfere with daily life. A global consensus in 2011 classified segmental vitiligo separately from all other types of vitiligo. The term vitiligo has been repurposed to refer to various types of nonsegmental vitiligo. There are numerous pharmaceutical procedures available on the market that aim to stop the development of and induce epidermal repigmentation. Variable levels of skin pigmentation have been observed with such therapies, either alone or in combination, and their predominance was safe and efficient. There are few vitiligo treatments available, and none of them can reliably cause repigmentation in every individual. Individualized management is required depending on geography, physical appearance, and the presence of illness activities. The preceding study aims to provide insight into the potential prospects of vitiligo medication while also summarizing the current body of knowledge on the condition.

## Introduction and background

Vitiligo is a depigmenting skin condition characterized by a specific melanocyte depletion, resulting in melanin attenuation inside the skin's damaged regions. A distinguishing feature is a completely amelanotic, non-scaly, chalky-white macule with clear borders [1]. The understanding of the etiology of vitiligo has advanced significantly in recent years. It is now categorically recognized as an autoimmune disorder associated with metabolism and oxidative stress, including cellular detaching diseases, as well as hereditary and environmental factors [1,2]. The consequences of vitiligo can be mentally distressing and frequently have a significant impact on daily life; thus, this should never be dismissed as an esthetic or minor illness [2]. The two main types of the condition recognized by a global consensus in 2011 were nonsegmental vitiligo (NSV) and segmental vitiligo (SV). The term "vitiligo" was chosen to refer to all NSV types (including acrofacial, mucosal, generalized, universal, mixed, and rare variants). One of the most important critical decisions made by this consensus was to distinguish SV from other types of vitiligo, especially given the implications for prognosis [2]. Vitiligo is still a common and identifiable condition among dermatologists, most doctors, and several wise members of the general population. The disease's defining feature is hypopigmented patches, which are frequently first noticed on the fingertips, knuckles, and area surrounding the lips, eyes, toes, and reproductive organs [3]. The two most common ways for the skin to turn white are as follows. Melanocytes produce melanin and then pack them into melanosomes that are transferred to the surrounding keratinocytes through their dendritic processes, which are then injected into neighboring keratinocytes. Keratinocytes transport melanins and melanosomes through the epidermis's basal layer to the stratum corneum, where cells are desquamated and released into the surrounding environment [4].

Certain diseases prevent or slow melanin production, causing the epidermis to become hypopigmented. Among these conditions are pityriasis alba, tinea versicolor, oculocutaneous albinism, and nevus depigmentosus. Melanocytes in the epidermis are generally present in healthy quantities; however, in several diseases, they produce less melanin than usual. Typically, the skin has mild to severe hypopigmentation [4]. The overall loss of melanocytes in vitiligo patients appears to be caused by three major factors. The argument is that people with vitiligo have three "vitiligo" alleles that predispose them to melanocyte degeneration. Because no two people can have the same three alleles, there are potentially many different combinations of three genes that can cause vitiligo. The second anomaly ultimately affects these melanocytes. Melanocytes from vitiligo patients differ from those from non-vitiligo patients [5]. Melanocytes from vitiligo patients, for example, require different and more exacting culture settings than those from healthy people.

Furthermore, vitiligo melanocytes were far more sensitive to phenolic compounds than regular melanocytes, and when exposed to them, they quickly experienced apoptosis [5]. The third component is an atmospheric element (or substance) that activates (or regulates) all relevant genes, resulting in the destruction of vulnerable melanocytes. The environmental factors that activate or suppress these vitiligo genes appear to cause an overly aggressive immunological response that causes melanocytes to die, resulting in skin hypopigmentation [5]. Melanocytes are the first cells to be affected by vitiligo. Keratinocytes, on the other hand, exhibit specific signs of injury, primarily grainy degradation. Merkel cells were never widely recognized as being present in depigmented skin [6]. It is unknown what its absence means or how it affects the epidermis's ability to function. Within the epidermis, keratinocytes, Langerhans cells, melanocytes, and Merkel cells work closely together. It could be assumed that if one or both of the epidermis's cells were lost, the epidermis's functionality would change [7]. It is thought that Merkel cells have neurosensory properties. It has been demonstrated that the hypopigmented skin of vitiligo patients has different sweating and bleeding patterns. Several researchers, but not all witnesses, had observed changes in the shape and cholinergic functioning of perspiration glands, which are normally influenced by cholinergic sympathetic nerves. Injury to collateral keratinocytes and Merkel cells may cause functional changes in hypopigmented skin [7,8]. The term "dermatomal," which refers to the arrangement of skin sensory nerves, is frequently confused with the adjective "segmental." Unilateral is a better phrase to avoid ambiguity because segmental is rarely, if ever, dermatomal. Vitiligo, whether bilateral or generalized, can begin at any age and often progresses sporadically over the course of a person's lifetime [8]. The resulting depigmentation is very symmetrical in its spread. A patch on the left half of your body corresponds to a patch on your right half. Depigmentation can occur throughout the body, but it is uncommon. Depigmentation usually appears in a symmetrical pattern, beginning with your fingers, toes, forearms, elbows, and upper arms and surrounding the lips and eyelids. Such symmetry is impossible to comprehend [8].

However, because vitiligo is so common, symmetrical depigmentation is a must in order to identify it. (Depigmented patches may appear irregularly dispersed. This is referred to as atypical vitiligo.) There are numerous significant differences between unilateral (segmental) vitiligo and generalized vitiligo. It usually begins in childhood or early adulthood; progresses for a short period of time, usually 1-2 years; and then remains stable for the rest of the person's life. Only one-half of your body is affected. In contrast to bilateral vitiligo, the dispersion on the skin is asymmetrical. However, these correlations are never arbitrary. Depigmentation affects a number of recurring areas [8]. The facial characteristics had been classified. In terms of position and physical appearance, the structures of the neck and torso mirror one another. In terms of appearance, texture, and placement, segmental vitiligo may resemble nevus depigmentosus and café-au-lait spots. These pattern similarities and differences between unilateral vitiligo and nevus depigmentosus suggest that melanocyte movement processes through the neural crest toward the epidermis during embryological maturation are comparable to those seen in unilateral vitiligo [9].

Methodology

The research was done utilizing internet searching engines, and thorough literary and information research was conducted. Articles retrieved after a search conducted in the PubMed database were included in the research. The search was carried out using keywords such as "depigmentation," "hypopigmentation," "vitiligo," "types of vitiligo," "types of hypopigmentation," and "types of depigmentation." A total of 210 articles were found, of which 30 were used for writing this narrative review.

## Review

Classification

Types of vitiligo and their subtypes are found in Table 1 [10].

**Table 1:** Types of vitiligo and their subtypes

Types of vitiligo	Subtypes
Nonsegmental vitiligo (NSV)	Focal (can evolve into segmental {SV} or nonsegmental vitiligo {NSV}), mucosal, acrofacial, generalized, universal, and rare variants of vitiligo (leukoderma punctata, hypochromic vitiligo, and follicular vitiligo)
Segmental vitiligo (SV)	Focal (can evolve into segmental {SV} or nonsegmental vitiligo {NSV}), unisegmental, bisegmental, or multisegmental
Mixed (NSV + SV)	Concomitant occurrence of SV and NSV according to the severity of SV
Unclassified	Focal at onset, multifocal, asymmetrical, nonsegmental, and mucosal (one site)

Diagnosis

The physical presence of developed, amelanotic, non-scaly, chalky-white macules with transparent edges in a characteristic dispersion in the mouth, tips of the lower extremity, genitalia, and segment and sites of friction usually yields an unambiguous identification of vitiligo [10]. Additional chemical testing is usually not required to establish vitiligo identification. A skin biopsy or additional testing is rarely required other than to rule out other illnesses. Non-invasive methods for determining whether a condition lacks melanocytes include in vivo confocal imaging and a skin sample [10]. According to the histopathology of a vitiligo patch's center, the epidermis's melanin pigmentation has completely disappeared, and no melanocytes are found [11]. Lymphocytes were only occasionally seen at the lesions' expanding edges. Portable ultraviolet (UV) illumination equipment that generates ultraviolet A (UVA), such as a Wood's lamp, could aid in the diagnosis of vitiligo. It aids in the destruction of localized melanocyte and detects regions of depigmentation that may never be visible to human sight, particularly in those with light skin [11]. Under Wood's light, the vitiligo spots glow brightly blue-white and have distinct borders. Dermoscopy was used to distinguish vitiligo from other depigmenting diseases. Several hypopigmentation syndromes lack residual perifollicular pigmentation and telangiectasia, which are typical vitiligo features.

Furthermore, it may aid in determining the stage of development and sickness behavior of vitiligo: Perifollicular pigmentation is seen in progressing lesions, whereas perifollicular depigmentation is seen in static or repatriating lesions [12]. Vitiligo can be diagnosed in a variety of ways. Hypopigmented areas resembling vitiligo can occur in a variety of common and unusual disorders. It is critical to distinguish vitiligo from melanoma-associated leukoderma and avoid misdiagnosis, primarily because it can occur before melanoma is discovered. Despite having a medically identical appearance, antibodies targeting the melanoma antigen recognized by T cells 1 (MART1) could distinguish melanoma-associated depigmentation from vitiligo [12]. Segmental hypopigmentation, also known as nevus depigmentosus, is usually present at birth or during the first year of life. Even though it may change as the child grows, it is consistent. Although nevi frequently have a healthy number of melanocytes with reduced melanin synthesis, it is a distinct differential diagnosis for SV. With Wood's light inspection, the difference between lesioned and healthy skin is less pronounced than in vitiligo [13].

Assessment of condition

A thorough preliminary evaluation is required for vitiligo hospital treatment. A thorough history and skin examination is required to assess vitiligo patients in order to determine the severity of the condition and any unique predictive variables. The Vitiligo European Task Committee developed an appraisal process that summarizes the medical diagnostic questions that could be useful for diagnosis [14]. Individuals must be questioned on a regular basis about their family history of thyroid illness, various autoimmune disorders, premature hair graying, vitiligo, and various skin conditions. The following elements must be considered: skin phototype; illness period; extensiveness; interaction; speed of progression or expansion of lesions; the presence of Koebner's phenomenon; the presence of halo nevi; prior therapies, including one's category, period, and performance; previous incidents of repigmentation; work-related history/exposure to toxins; and the impact of the illness on one's standard of living [15]. Certain body parts are especially vulnerable to Koebner's phenomenon due to typical behaviors such as cleanliness, attire, and employment. It might be possible to get rid of vitiligo by looking for Koebner's phenomenon, which causes vitiligo after physical damage [16]. The Koebner's phenomenon in vitiligo score (K-VSCOR) was developed and validated as a rating system for assessing the likelihood of Koebner's phenomenon. Individuals who scored highly must be advised on how to avoid physical strain.

Numerous studies have found links between vitiligo and autoimmune diseases such as thyroid issues, alopecia areata, rheumatoid arthritis, adult-onset diabetes, Addison's disease, pernicious anemia, systemic lupus erythematosus, psoriasis, and atopic dermatitis [16]. Thyrotropin levels must be monitored on a regular basis, especially in patients who have antibodies against thyroid peroxidase at the preliminary examination, because NSV increases the risk of autoimmune thyroid disease, particularly Hashimoto's thyroiditis. Antibodies against thyroid peroxidase must be tested. Individuals with vitiligo are more susceptible to autoimmune diseases based on their cultural background and family history. When organ-specific autoimmune disorders are present, their signs and symptoms must prompt an appropriate evaluation and professional advice. Koebner's phenomenon, trichrome lesions, inflammatory lesions, and confetti-like depigmentation are the most obvious and well-studied medical signs of a rapidly progressing illness [17]. The observed intensity of the vitiligo and an individual's mindset were indicators of the comfort of living deterioration; thus, a comprehensive evaluation of psychological traits and ease of living is required. A standardized vitiligo quality-of-life scale has been developed. Every vitiligo patient must receive psychological counseling and support [17].

Management

The most difficult dermatological problem is probably the constant treatment of vitiligo. Recognizing that vitiligo is more than just a cosmetic condition and knowing that effective treatments are available are critical first steps in managing the disease [18]. Surgery, local and oral immunosuppressive agents, and phototherapy may all work together to slow the progression of the condition, stabilize hypopigmented lesions, and promote repigmentation. The classification of the illness and its scope, dispersion, and activities, as well as the person's age, phototype, impact on quality of life, and desire for therapy, all influence the medication decision [19]. While the mouth and distal limbs are resistant to treatment, the face, throat, torso, and mid-extremities are the most responsive. Repigmentation occurs around the edges of lesions or in a perifollicular pattern. To determine the effectiveness of a treatment, it must be used for at least two to three months. UV light-based treatments, the most popular vitiligo medication, have been linked to a better outcome when combined with another medication [20]. Treatment necessitates an individualized pharmacological methodology in which individuals must be constantly addressed because most treatment options are time-consuming and require lengthy follow-up. Individuals with vitiligo in visible areas must seek esthetic concealment advice from a licensed cosmetologist or a trained nurse. Self-tanning creams containing dihydroxyacetone that provide long-lasting color for up to several weeks, as well as foundation-based beauty solutions, are examples [21]. The Vitiligo Subcommittee of the European Dermatology Forum has produced standards for the diagnosis and treatment of vitiligo based on the most robust available research and professional advice. The medicines were ranked from first- to fourth-line options. Topical therapies (corticosteroids and calcineurin inhibitors) are first-line treatments. The two second-line therapies are phototherapy (narrow-band ultraviolet B {NB-UVB} and psoralen plus UVA {PUVA}) and systemic steroid management. The third and fourth lines of treatment are surgical grafting methods, including depigmenting therapies [22].

Pharmacological management

Topical Treatment Corticosteroids

Corticosteroids have a significant medicinal impact in vitiligo by regulating and suppressing the inflammatory response. Topical corticosteroids (TCS) are the first-line treatment for vitiligo, whether potent (betamethasone valerate) or highly potent (clobetasol propionate). The therapeutic effects are stronger in sun-exposed areas, whereas acral zones typically produce poor results [23].

Calcineurin Inhibitors

Topical calcineurin inhibitors (TCIs) targeting the head and neck region include tacrolimus (0.03% or 0.1%) and pimecrolimus (1%) [18,21], which have fewer adverse effects, particularly no risk of atrophy. For at least six months, TCI could be used twice a day. The course of therapy can be extended if there are visible positive outcomes. During treatment, moderate daily sun exposure is advised [24].

Vitamin D3 Analogues (D3A)

Topical vitamin D3 analogues (D3A) are not effective as a stand-alone treatment for vitiligo due to their immunomodulatory properties, which decrease T cell function, promote melanocyte formation, and induce melanogenesis. Nonetheless, they are useful as supplements to other treatments. The optimum dosage for four weeks when applying the ointment and eight weeks when applying the cream is 100 g weekly on 30% of the body area, plus a combination of calcipotriol 0.005% and betamethasone 0.05% [25].

5-Fluorouracil (5-FU); methotrexate (MTX); prostaglandin F2 alpha analogues, a peptide derived from primary basic fibroblast growth factor (bFGF); inhibitors of Janus kinase (JAK), systemic therapy corticosteroids; apremilast; etc. are some other promising pharmacological treatments that include minocycline antibiotic use [25-27].

Physical therapy

Narrow-Band UVB Phototherapy

UV irradiation appears to have several systemic effects, including stimulation of the central hypothalamic-pituitary-adrenal axis, initiation of the proopiomelanocortin route in the hypothalamic arcuate nucleus, immunosuppressive effects, and opioid genic outcomes. UVB (wavelength of 280-320 nm) irradiance is more prominent than UVA (wavelength of 320-400 nm) [27]. NB-UVB photodynamic therapy (wavelength of 311 nm) suppresses the immune system, induces melanocyte separation, increases melanin synthesis, and causes melanocyte emigration from perilesional skin to treat vitiligo [28].

PUVA

PUVA irradiation (wavelength of 320-340 nm) causes melanin production by suppressing the immune system and fostering an environment conducive to the formation of melanocytes. This second-line treatment usually involves applying psoralen topically or ingesting it and then exposing it to UVA. Psoralens are taken orally for 1-3 hours before UVA exposure [29]. Other physical management techniques in use include combined Fraxel Erbium and UVA1 lasers and laser therapy excimer laser (EL) [30].

## Conclusions

Vitiligo is a multivariate skin condition with a complicated pathophysiology. Despite recent significant advances in human knowledge of this condition, the origin and pathophysiology of vitiligo remain unknown. There are still questions about what causes melanocyte degeneration, and more research is needed to fully understand the etiology of vitiligo. It is critical to understand the biological messengers and molecular processes that result in metabolic abnormalities, melanocyte destruction, and autoimmune disorders to find novel treatment objectives and medications that may arrest the spread of the illness and possibly treat vitiligo. Natural cytokine-targeting treatments have been shown to be effective in treating conditions such as psoriasis and vitiligo. As a result, attacking the interferon (IFN)-chemokine axis with current or future medicines is appealing and intriguing. The inconsistent therapeutic progress and recurring nature of vitiligo medication can be discouraging at times. Customized treatment plans must be developed based on the type of vitiligo, whether it is active, and the side effects of the drug. There are only a few vitiligo treatments available, and none of them can reliably cause repigmentation in every individual. Additional scientific and therapeutic research is required to develop new therapeutic strategies and gain a better understanding of the vitiligo etiology. Many new medicines are on the horizon, and the majority of information about them is provided by case studies or episodes. Additional randomized controlled trials are required to accurately assess their effectiveness.
